# *Caenorhabditis elegans* susceptibility to gut *Enterococcus faecalis* infection is associated with fat metabolism and epithelial junction integrity

**DOI:** 10.1186/s12866-016-0624-8

**Published:** 2016-01-15

**Authors:** Shuzhen Sim, Martin L. Hibberd

**Affiliations:** Infectious Diseases, Genome Institute of Singapore, 60 Biopolis Street, #02-01 Genome, Singapore, 138672 Singapore; Faculty of Infectious and Tropical Diseases, London School of Hygiene and Tropical Medicine, Keppel Street, London, WC1E 7HT United Kingdom

**Keywords:** Gut microbiome, Host-microbe interactions, Nuclear receptors, Fat metabolism, Epithelial junction integrity

## Abstract

**Background:**

Gut bacteria-host interactions have been implicated in the pathogenesis of numerous human diseases, but few mechanisms have been described. The genetically tractable nematode worm *Caenorhabditis elegans* can be infected with pathogenic bacteria, such as the human gut commensal *Enterococcus faecalis,* via feeding, making it a good model for studying these interactions.

**Results:**

An RNAi screen of 17 worm candidate genes revealed that knockdown of the transcription factor *nhr-49*, a master regulator of fat metabolism, shortens worm lifespan upon infection with *E. faecalis* (and other potentially pathogenic bacteria) compared to *Escherichia coli*. The functional similarity of *nhr-49* to the mammalian peroxisome proliferator-activated receptors (PPARs) suggests that this is mediated through a link between fatty acid metabolism and innate immunity. In addition, knockdown of either *dlg-1* or *ajm-1*, which encode physically interacting proteins in the *C. elegans* epithelial junction, also reduces worm lifespan upon *E. faecalis* challenge, demonstrating the importance of the intestinal epithelium as an immune barrier.

**Conclusions:**

The protective roles identified for *nhr-49*, *dlg-1*, and *ajm-1* suggest mechanistic interactions between the gut microbiota, host fatty acid metabolism, innate immunity, and epithelial junction integrity that are remarkably similar to those implicated in human metabolic and inflammatory diseases.

**Electronic supplementary material:**

The online version of this article (doi:10.1186/s12866-016-0624-8) contains supplementary material, which is available to authorized users.

## Background

The human gastrointestinal tract harbors an enormous number and variety of microbes, with a collective genome size estimated at ~150 times the complement of human genes [1]. These organisms, collectively termed the gut microbiome, carry out crucial metabolic and biological functions. They aid in food digestion and nutrient uptake, play important roles in the development of the immune system and intestinal epithelial barrier, and compete with potentially pathogenic microorganisms, thus curbing their growth (reviewed in [2, 3]). Studies of germ-free or gnotobiotic mice have shown that these microbes also have local and systemic effects outside of the gut [4–8], highlighting the complex nature of their interactions with the host, although the mechanisms of these interactions require further research.

Disturbances of the gut microbiome through factors such as diet, antibiotic treatment, or pathogen invasion can lead to dysbiosis, in which there is an outgrowth of potentially pathogenic bacteria and a decrease in beneficial bacteria. The consequent alteration in host interactions is thought to result in a perturbed immune balance, which has been linked to the pathogenesis of diverse intestinal and systemic human diseases (reviewed in [9–11]). These include inflammatory bowel diseases (IBD) such as ulcerative colitis and Crohn’s disease [12–15], and metabolic disorders or conditions such as metabolic syndrome, obesity, and type 2 diabetes (T2D) [16–19].

Further evidence comes from the perspective of human genetics (reviewed in [20]). Genome-wide association studies (GWAS) have identified loci associated with susceptibility to IBD [21–23] and T2D [24, 25] that are also associated with microbial defense, thus supporting a role for host-microbiome interactions in disease pathogenesis. For example, *NOD2,* the first gene to be associated with IBD, encodes an intracellular pattern recognition receptor that mediates the host immune response to the bacterial peptidoglycan product muramyl dipeptide (MDP) [26]; other IBD-associated genes with roles in anti-bacterial immunity include *CARD9, IL23R*, and *LRRK2* [21–23]. Interestingly, there is considerable overlap between risk loci for Crohn’s disease and leprosy [27–29], suggesting a potential mycobacterial component to the pathogenesis of IBD. However, studies that have reported links between microbial dysbiosis, host genetics, and disease often lack direct causal or functional evidence.

To address this lack of functional evidence, we have utilized the genetically tractable nematode worm *Caenorhabditis elegans*, a commonly used model for understanding microbial host-pathogen interactions [30–33]. Since *C. elegans* is a bacterivore, intestinal infection can be achieved through feeding, making it especially ideal for investigating interactions with gut bacteria. In this study, to identify potentially pathogenic host-microbiome interactions, we selected nematode candidate genes whose orthologs or functionally related genes in the human have previously been implicated in metabolic or inflammatory diseases. An RNAi screen of these genes identified two aspects of nematode biology—fatty acid metabolism and epithelial junction integrity—that are involved in *C. elegans* defense against the human gut commensal *Enterococcus faecalis,* an opportunistic pathogen commonly associated with hospital-acquired, multidrug-resistant infections [34].

## Results

### *Knockdown of* nhr-49 *or* dlg-1 *reduces nematode lifespan upon OG1RF challenge*

OG1RF, an *E. faecalis* strain of human origin, kills *C. elegans* by establishing a persistent infection in the nematode intestine, where it proliferates extensively from a small initial inoculum and causes gross distention [31]. The thick cell wall of *E. faecalis* is thought to protect it against the *C. elegans* grinder organ, which normally disrupts ingested bacteria [31].

To identify host-bacteria interactions during *E. faecalis* infection of *C. elegans,* we performed RNAi knockdowns of 17 candidate *C. elegans* genes (Additional file 1: Table S1), screening for impact on nematode survival upon OG1RF challenge. These candidates have functional domains that suggest potential interactions with bacteria, but had not previously been characterized in the context of microbial infection in the nematode. In addition, their human orthologs or functionally related genes have been implicated in metabolic and inflammatory diseases.

**Fig. 1:**
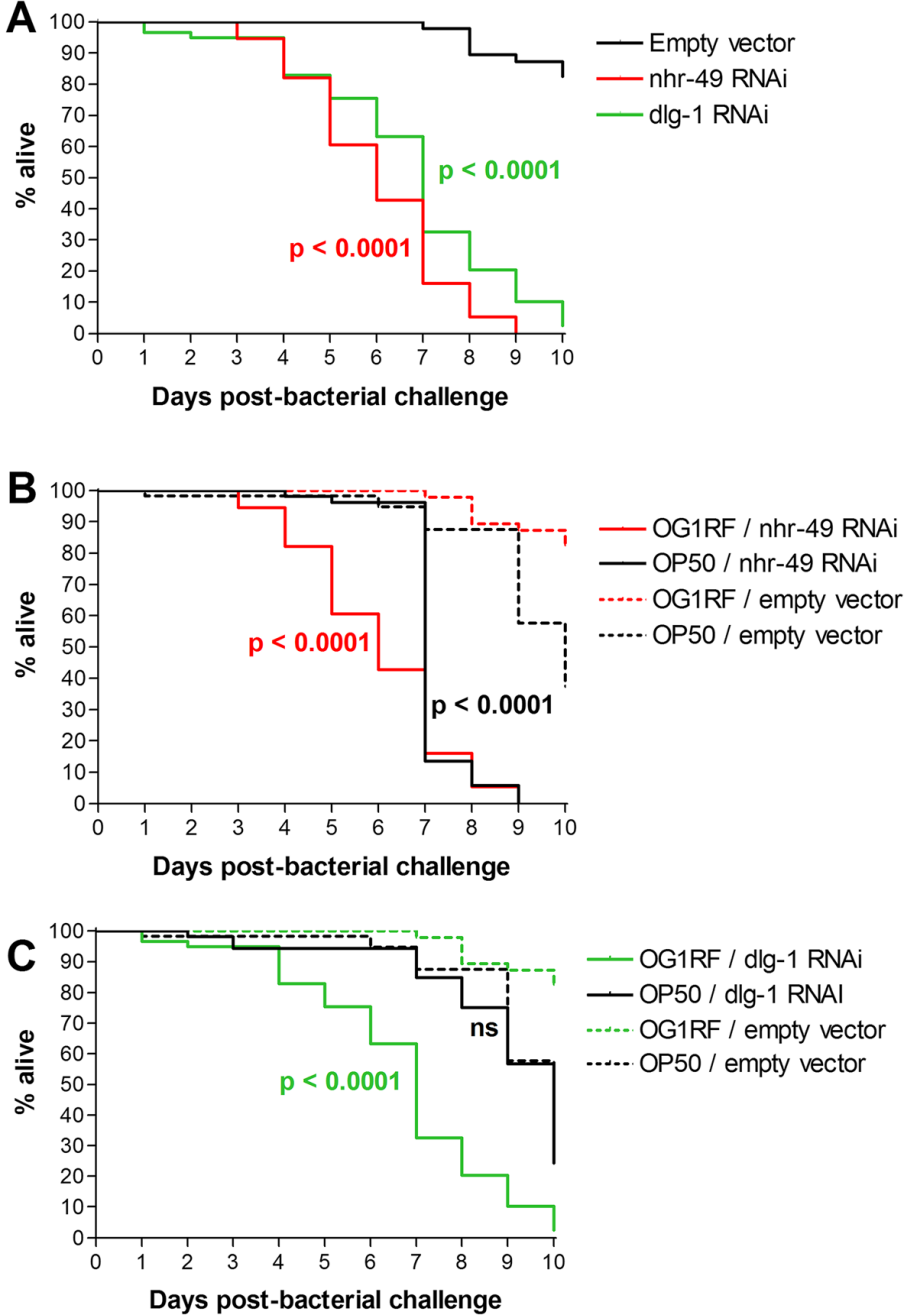
Effect of gene knockdown on *C. elegans* lifespan upon challenge with OG1RF. **a** Kaplan-Meier survival curves showing the effect of *nhr-49* and *dlg-1* knockdown upon challenge with OG1RF. **b**-**c** Data from (**a**) shown alongside survival curves for *nhr-49* and *dlg-1* knockdown upon challenge with OP50; OG1RF and OP50 survival assays were performed on separate occasions. Data represent three biological replicates with 20–30 nematodes each; *p*-values indicate comparisons between gene knockdown and empty vector for the same bacteria

Knockdown of two candidate genes—*nhr-49* and *dlg-1*—resulted in a significant decrease in nematode lifespan upon exposure to OG1RF, compared to the RNAi vector control (Fig. 1a). As has been previously reported [35], knockdown of *nhr-49* on OP50 also resulted in a significant decrease in lifespan (Fig. 1b). However, this reduction (from a median of 10 days to 7 days) was not as severe as compared to knockdown on OG1RF (from undefined, i.e. end-point survival exceeded 50 %, to 6 days) (p < 0.0001) (Fig. 1b). Knockdown of *dlg-1* on OP50 had no effect on lifespan (Fig. 1c).

### nhr-49 *and* dlg-1 *impact* C. elegans *interactions with other bacteria*

**Fig. 2:**
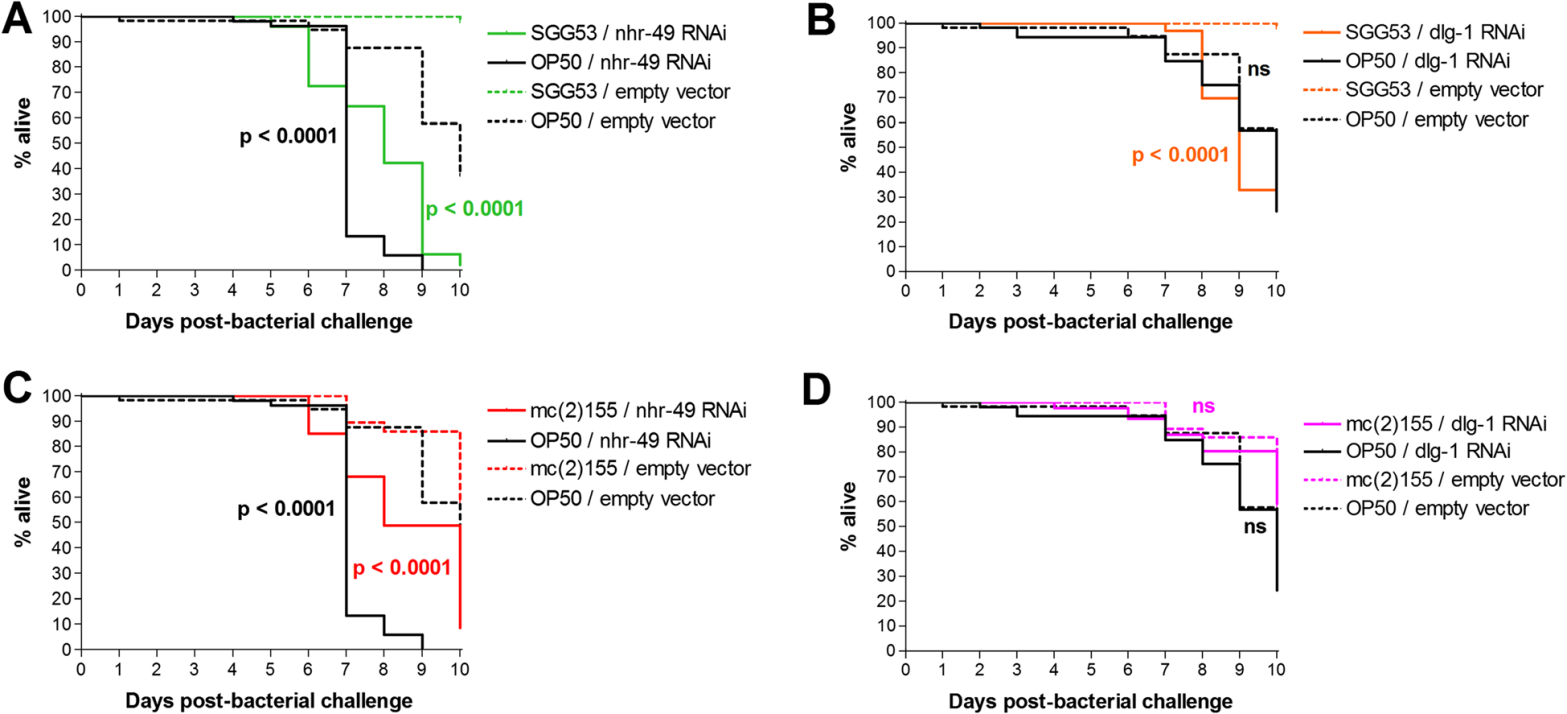
Effect of gene knockdown on *C. elegans* lifespan upon challenge with other bacteria. Kaplan-Meier survival curves showing effect of *nhr-49* and *dlg-1* knockdown upon challenge with (**a**-**b**) SGG53 and (**c**-**d**) mc(2)155. Survival curves for gene knockdowns on OP50, performed on a separate occasion, are also shown. Data represent three biological replicates with 20–30 nematodes each; *p*-values indicate comparisons between gene knockdown and empty vector for the same bacteria

We also examined the effect of *nhr-49* and *dlg-1* knockdown on nematode lifespan upon challenge with other potentially pathogenic bacteria species. *Streptococcus gallolyticus gallolyticus* (previously classified as *S. bovis*) is a Gram-positive opportunistic human pathogen that has consistently been associated with colorectal cancer, although a direct causal link has yet to be established [36, 37]. Knockdown of *nhr-49* or *dlg-1* on SGG53, a clinical *S. gallolyticus gallolyticus* isolate, also resulted in a significant reduction in nematode lifespan (Fig. 2a and b).

The non-pathogenic, fast-growing *Mycobacterium smegmatis* strain mc(2)155 is often used as a laboratory model of mycobacterial disease. GWAS have reported an overlap between susceptibility genes for leprosy (caused by *Mycobacterium leprae*) and Crohn’s disease [27–29]; *Mycobacterium avium paratuberculosis* has also been linked to Crohn’s (reviewed in [38]). While these studies hint at a potential mycobacterial component to the pathogenesis of IBD, this has not been substantiated with direct evidence. Here, knockdown of *nhr-49* on mc(2)155 also significantly reduced nematode lifespan (Fig. 2c), whereas *dlg-1* knockdown on mc(2)155 showed no effect (Fig. 2d).

Because median lifespans for nematodes maintained on SGG53 and mc(2)155 were undefined, we could not tell if the lifespan reduction seen upon *nhr-49* knockdown on these bacteria was more or less severe than for knockdown on OP50 (Fig. 2a and c).

Taken together, the data suggest that *nhr-49* affects interactions with OG1RF, SGG53, and mc(2)155, with the greatest impact on OG1RF, while *dlg-1* affects interactions with OG1RF and SGG53.

### *Knockdown of* nhr-49 *transcriptional targets does not impact survival on OG1RF*

**Fig. 3:**
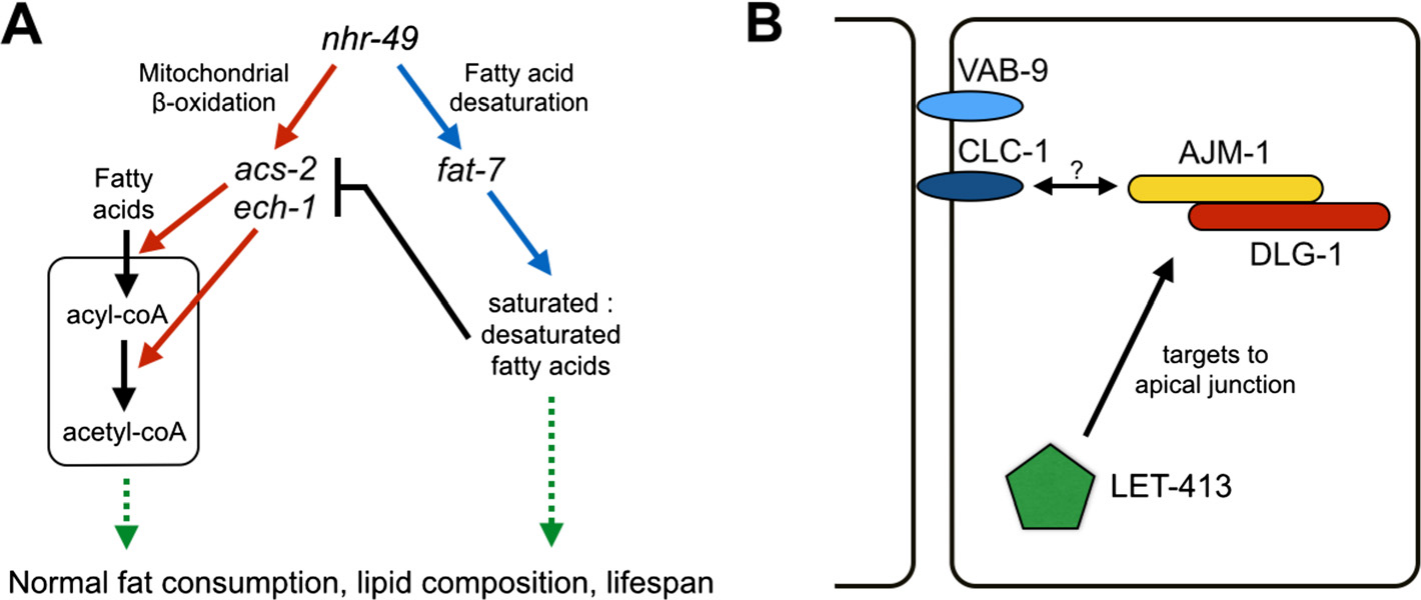
Modes of action of *nhr-49* and *dlg-1*. **a** *nhr-49* encodes a nuclear hormone receptor that enhances the expression of *acs-2* and *ech-1*, facilitating the flow of fatty acids into the mitochondrial matrix. *nhr-49* also maintains a normal balance of saturated and unsaturated fatty acids by enhancing expression of *fat-7*. Deletion or depletion of *nhr-49* increases fat content and reduces lifespan in *C. elegans* maintained on OP50. *fat-7* is involved in a negative feedback mechanism that inhibits *acs-2* and *ech-1* expression, possibly by signaling through an as-yet unidentified fatty acid species. **b** *dlg-1* encodes a protein that physically interacts with *ajm-1* in the *C. elegans* epithelial junction. The DLG-1 / AJM-1 complex is targeted to apical junctions by LET-413, and may be linked to the cell membrane by claudin-like transmembrane proteins such as VAB-9 or CLC-1. See text for more details; figures have been adapted from [35, 39]

*nhr-49* encodes a nuclear hormone receptor master regulator of fat metabolism. Loss of *nhr-49* increases fat content and reduces lifespan in nematodes maintained on OP50 [35]. *nhr-49* enhances expression of *acs-2,* a mitochondrial acyl-CoA synthetase, and *ech-1,* a mitochondrial β-oxidation trifunctional enzyme, thus promoting the flow of fatty acids into the mitochondria for use in energy production. *nhr-49* also enhances expression of *fat-7,* a stearoyl-CoA desaturase, thus maintaining proper balance of saturated to unsaturated fatty acids. *fat-7* is involved in a negative feedback mechanism that inhibits *acs-2* and *ech-1* expression, possibly by signaling through an as-yet-unidentified fatty acid species [35] (Fig. 3a).

**Fig. 4:**
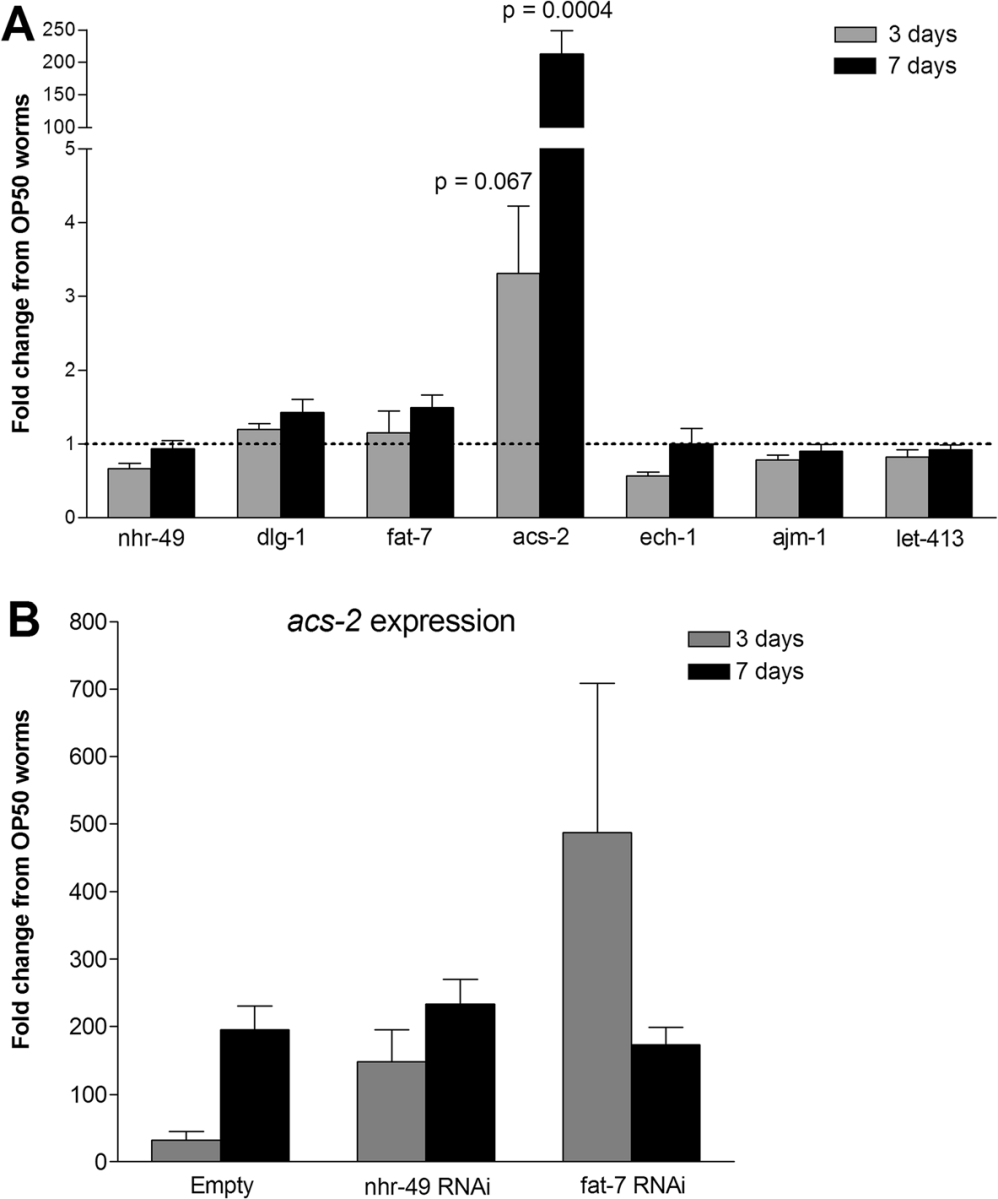
Effect of OG1RF challenge on *C. elegans* gene expression. **a** Fold change in gene expression at 3 and 7 days post-OG1RF challenge, relative to nematodes grown on OP50. **b** Fold change in *acs-2* expression upon gene knockdown, at 3 and 7 days post-OG1RF challenge, relative to gene knockdown on OP50

We examined the effect of OG1RF challenge on *nhr-49*, *acs-2*, *ech-1*, and *fat-7* expression by quantitative RT-PCR. By 7 days post-OG1RF challenge, the transcript abundance of *acs-2* was induced ~200-fold (*p* = 0.0004) relative to nematodes maintained on OP50 (Fig. 4a).

We next determined if OG1RF induction of *acs-2* was dependent on *nhr-49* or *fat-7*. Knockdown of *nhr-49* resulted in a moderately stronger induction of *acs-2* upon OG1RF challenge (222-, 163-, and 56-fold versus 54-, 8, and 33-fold relative to worms on OP50) (Fig. 4b). Knockdown of *fat-7* resulted in a much more appreciable and consistent induction of *acs-2* (178-, 368-, and 916-fold versus 54-, 8-, and 33-fold relative to worms on OP50) (Fig. 4b). However, due to the large range of the data, a student’s t-test did not find these changes statistically significant. These data underscore the role of *fat-7* as a negative regulator of *acs-2*, and suggest that *nhr-49* depletion and subsequent reduced *fat-7* transcription may result in moderate loss of this regulation. The relative balance between *nhr-49* and *fat-7* levels are likely to contribute to the variability in the data.

**Fig. 5:**
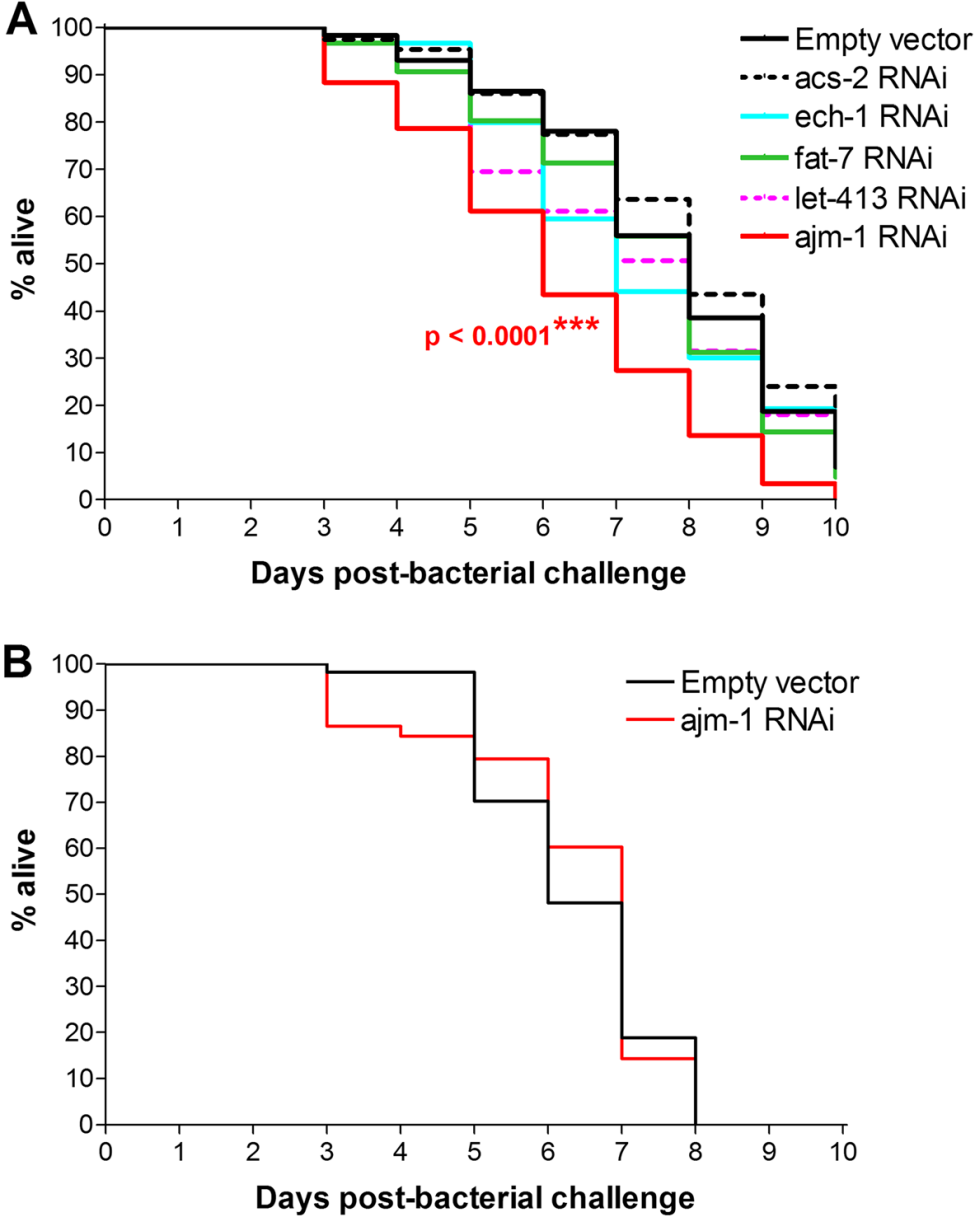
Effect of knockdown of *nhr-49*- and *dlg-1*-interacting genes on *C. elegans* lifespan upon challenge with OG1RF. **a** Kaplan-Meier survival curves showing effect of gene knockdown upon challenge with OG1RF. **b** Kaplan-Meier survival curves showing effect of *ajm-1* knockdown on OP50

Despite its strong induction by OG1RF, knockdown of *acs-2* had no effect on nematode lifespan after bacterial challenge. Similarly, knockdown of either *fat-7* or *ech-1* separately did not impact lifespan after bacterial challenge (Fig. 5a).

### *Knockdown of the* dlg-1*-interacting gene* ajm-1 *shortens lifespan on OG1RF*

*dlg-1* encodes a protein that physically interacts with AJM-1 in the *C. elegans* epithelial junction. The DLG-1 / AJM-1 complex is targeted to apical junctions by LET-413 (Fig. 3b) (reviewed in [39]).

OG1RF infection did not modulate *dlg-1*, *ajm-1*, or *let-413* expression (Fig. 4a). Knockdown of *ajm-1* resulted in a significant decrease in lifespan for nematodes maintained on OG1RF (Fig. 5a) but not on OP50 (Fig. 5b); knockdown of *let-413* did not impact lifespan on OG1RF (Fig. 5a).

## Discussion

Insights from human GWAS and studies of germ-free animals suggest that host-microbiome interactions may play roles in triggering inflammatory and metabolic diseases (reviewed in [11, 20]). However, as causative organisms and disease mechanisms have yet to be definitively identified, this concept remains largely speculative. To address this lack of functional evidence, we used the *C. elegans* model system to identify potentially pathogenic host-microbe interactions. We identified two aspects of host physiology—fat metabolism and epithelial junction integrity—that *E. faecalis* interacts with during the killing of *C. elegans.*

Depletion of *nhr-49*, which encodes a nuclear hormone receptor and master regulator of fat metabolism, shortens worm lifespan upon *E. faecalis* infection. *nhr-49* shares strong sequence similarity with the mammalian hepatocyte nuclear factor 4 (HNF4) receptors; mutations in HNF-4A are associated with maturity onset diabetes of the young 1 (MODY1) and T2D (reviewed in [40]). However, *nhr-49*′s effects on fat metabolism and lifespan are more closely analogous to those of the mammalian peroxisome proliferator-activated receptors (PPARs) [35]. PPARs—nuclear hormone receptor transcription factors activated by fatty acids and their derivatives—are key regulators of metabolism and controllers of inflammation (reviewed in [41]), and are promising targets for the treatment of metabolic syndrome and IBD [42–44].

Obesity, insulin resistance, and other metabolic syndrome-related conditions are characterized by chronic, low-grade inflammation in metabolic tissues such as adipose tissue, liver, skeletal muscle, and blood vessel walls [11, 41]. Studies in mice have found that gut bacteria-derived lipopolysaccharide (LPS) can initiate this inflammatory state, and consequently obesity and insulin resistance, by binding to CD14-TLR4 complexes on immune cells [45, 46]. High fat diets can cause changes in the gut microbiota composition that result in increased circulating LPS levels [45].

PPARs control inflammation through multiple mechanisms (reviewed in [41]). In adipose tissue and the liver, the presence of alternatively activated macrophages (AAMs), which dampen inflammation and improve insulin sensitivity, is maintained by PPAR-dependent mechanisms [47, 48]. PPARs also reduce inflammation in muscle cells by regulating saturated fatty acid metabolism away from diacylglycerol accumulation, thus avoiding NF-ĸB activation [49].

PPARs also regulate inflammation in the intestinal epithelium, and there is ample evidence for more direct, local interactions between these molecules and gut bacteria. For example, the human gut commensal *Bacteroides thetaiotaomicron* enhances complex formation between PPARγ and the NF-ĸB subunit RelA in human intestinal cell lines, resulting in PPARγ-dependent nuclear export of RelA and subsequent suppression of pro-inflammatory cytokine production [50]. In addition, *E. faecalis* isolated from newborn babies induces phosphorylation and activation of PPARγ in colonic epithelial cell lines and primary colonic cells, resulting in transcriptional activation of target genes, including the immune-modulatory cytokine IL-10 [51]. These studies suggest potential links between fatty acid signaling / metabolism and innate immunity [41], and, similar to what we observe here for *nhr-49,* a protective role for PPARγ in gut homeostasis.

In our study, *E. faecalis* infection strongly induced expression of the *nhr-49* transcriptional target *acs-2*, an acyl-CoA synthetase that activates fatty acids for β-oxidation and energy production in the mitochondria. Both pathogenic and commensal bacteria, including *E. faecalis*, have been reported to regulate host energy production pathways, presumably to increase levels of metabolites required for their own growth [52–54]. However, *acs-2* depletion had no effect on nematode lifespan upon bacterial challenge, suggesting that *acs-2*-dependent metabolites may not be limiting to *E. faecalis* growth. Consistent with this, depletion of the *acs-2* negative regulator *fat-7* also did not impact lifespan upon *E. faecalis* challenge, despite a marked increase in *acs-2* induction. These data suggest that the protective effect of *nhr-49* may be mediated through one or more of its other transcriptional targets [35], which may have as-yet uncharacterized roles in innate immunity. Alternatively, analogous to what has been reported for PPARγ and RelA [50], this effect could also be mediated through a physical interaction between NHR-49 and other nuclear hormone receptor family members [55]. Finally, although the vast majority of *nhr-49* transcriptional targets play roles in fat metabolism [35, 56, 57], the possibility of OG1RF sensitivity being mediated by targets that affect other biological processes remains open.

Depletion of *dlg-1* or *ajm-1* also resulted in reduced worm lifespan upon *E. faecalis* challenge. The DLG-1 / AJM-1 complex is a critical component of the *C. elegans* apical epithelial junction, which, as in other organisms, mediates cellular permeability, adhesion between neighboring cells, and cell polarity (reviewed in [39]). Loss of either protein results in developmental arrest at the embryonic elongation stage [58, 59]: Loss of *dlg-1*, which encodes a Membrane Associated GUanylate Kinase (MAGUK) protein family member, results in disappearance of the electron-dense apical junction structure in epidermal and intestinal epithelia, as well as mislocalization of AJM-1 [58]. Loss of *ajm-1*, encoding a novel protein with a large coiled-coil domain, produces bubble-like separations at cell-cell junctions that are normally in close apposition [59]. Here, we show that depletion of *dlg-1* or *ajm-1* from the L1 stage onwards affects the susceptibility of adult worms to bacteria, suggesting that epithelial junction integrity constitutes an important gut defense mechanism.

Although the role of DLG-1 / AJM-1 in apical junction integrity is well-characterized, a link between this and intestinal permeability remains to be demonstrated. This may be mediated through the claudin CLC-1, which is thought to interact with DLG-1 / AJM-1 at the cell surface—CLC-1-deficient nematodes show compromised barrier function in the pharyngeal portion of the intestine, and leak ingested dye into the body cavity [60]. We hypothesize, however, that increased sensitivity to bacteria-derived toxins or small molecules (rather than dissemination of entire bacterial cells beyond the intestine) may be sufficient to bring about increased pathogen susceptibility.

The role of *dlg-1* in the maintenance of the intestinal epithelial immune barrier appears to be conserved in *Drosophila*, where depletion of its fly homolog *discs large (dlg*), a key component of the fly epithelial junction, results in over-proliferation of intestinal cells upon *Pseudomonas aeruginosa* infection [61]. Loss of other *Drosophila* epithelial junction components has also been reported to compromise gut defenses; notably, loss of the *big bang (bbg)* gene, which encodes a membrane-associated PDZ (PSD-95, Discs-large, ZO-1) domain-containing protein, loosens epithelial junctions, allowing intestinal bacteria to constitutively activate fly midgut immune responses. The resulting chronic epithelial inflammation reduces lifespan [62].

While not fully understood, human IBD is also thought to stem from a breach in the intestinal epithelium, which exposes immune cells in the underlying lamina propria to bacteria or bacterial products from the gut (reviewed in [63]). Genetic variants in DLG5 (a human homolog of *C. elegans dlg-1* and *Drosophila dlg*) are associated with IBD [64, 65], and intestinal permeability has been correlated with peripheral immune activation and clinical relapse in patients with Crohn’s disease [66, 67]. In IL-10 knockout mouse models of IBD, an unchecked T_H_1 inflammatory response is associated with increased intestinal permeability [68]; other mouse models indicate that disruption of intestinal epithelial junction integrity alone, through expression of dominant negative E-cadherin, is sufficient to initiate the disease [69]. In addition, consistent with the concept of gut bacteria-initiated systemic inflammation, increased intestinal permeability has been associated with metabolic conditions such as obesity, increased visceral adiposity, and fatty liver, both in patients and in animal models [70, 71].

## Conclusions

We identified protective roles for *nhr-49*, *dlg-1*, and *ajm-1* in *C. elegans* defense against the human gut commensal *E. faecalis.* The involvement of these genes suggests intriguing interactions between gut bacteria and the host processes of fatty acid metabolism, innate immunity, and epithelial junction integrity that consequently impact nematode survival. These data lend mechanistic support to the concept of gut microbiota dysbiosis in humans, where remarkably similar interactions have been proposed as a basis for the pathogenesis of inflammatory and metabolic diseases.

## Methods

### Nematode and bacteria strains

The *C. elegans* strain SS104 *glp-4(bn2)I*, a temperature-sensitive mutant that grows permissively at 15 °C but produces no progeny at 25 °C [72], was used in survival assays. This was maintained at 15 °C on nematode growth media (NGM) agar and fed on the OP50 *Escherichia coli* strain, as described previously [73].

*E. coli* OP50 was grown at 37 °C in Luria-Bertani (LB) broth. OG1RF (ATCC accession number 47077), an *Enterococcus faecalis* strain of human origin, was grown in brain-heart infusion (BHI) broth (BD) at 37 °C. SGG53, a *Streptococcus gallolyticus gallolyticus* clinical isolate, was grown at 37 °C in tryptic soy (TS) broth (BD). mc(2)155 (ATCC accession number 700084), a *Mycobacterium smegmatis* strain, was grown at 37 °C in Middlebrook 7H9 broth supplemented with ADC enrichment (BD).

### RNAi knockdown of candidate genes

Nematodes were fed on HT115 (DE3) *E. coli* clones transformed with either the empty L4440 vector (control), or the L4440 vector containing a gene-specific amplicon. RNAi constructs were either obtained from the Ahringer RNAi library [74] or created by cloning gene-specific amplicons into L4440 vector. Sequences of primers used for PCR amplification are listed in Additional file 2: Table S2.

Each clone was grown for 8 h in LB broth supplemented with 50 μg/ml ampicillin, seeded on NGM agar plates supplemented with 25 μg/ml carbenicillin and 1 mM β-D-1-thiogalactopyranoside (IPTG), and left to induce overnight at room temperature. Nematode embryos, generated by hypochlorite treatment, were grown on these plates until the L4 stage, and subsequently transferred to pathogen lawns.

### Survival assays

Pathogens were grown overnight in their respective liquid media at 37 °C, seeded onto their respective agar plates, and left to form lawns overnight at 37 °C. To minimize transfer of HT115 *E. coli*, L4-stage nematodes were allowed to crawl on bare agar before they were transferred to pathogen lawns. Subsequently, nematodes were transferred to fresh pathogen lawns every other day to further prevent growth of residual HT115 *E. coli*; none was visually observed. Nematode survival was monitored daily; nematodes were considered dead if they failed to respond to gentle touch with a platinum wire. The data presented are a pool of three independent experiments, with 20–30 nematodes each. Survival assays for OP50 and for each bacterial pathogen were performed on separate occasions; however, statistical comparisons presented in the Figures were made only within the same assay.

### Quantitative real-time PCR analysis

Nematodes were collected in 1 ml Trizol, and lysed by freeze-thawing. RNA was extracted from lysates with the Qiagen RNeasy Mini Kit (Qiagen), DNAse-treated (ThermoScientific), and used for cDNA synthesis with the Maxima H Minus First Strand cDNA Synthesis Kit (ThermoScientific). qRT-PCR was performed with the KAPA SYBR FAST qPCR Master Mix (KAPA Biosciences) on the LightCycler 480 II real-time thermocycler (Roche Applied Science). Raw values were normalized to each of three control genes (*act-1, nhr-23,* and *ama-1*); the average of the normalized values was then used for analysis [75]. Primer sequences used in qRT-PCR are presented in Additional file 3: Table S3.

### Statistical analysis

Kaplan-Meier survival curve analysis and student t-tests were carried out with GraphPad Prism 3.

## Additional files

Additional file 1: Table S1.
*C. elegans* candidate genes screened via RNAi knockdown. IBD, inflammatory bowel disease; T2D, type 2 diabetes; KD, Kawasaki Disease. (DOCX 15 kb) Additional file 2: Table S2. Sequences of primers used for PCR amplification of gene-specific amplicons for cloning into the L4440 RNAi vector. RNAi constructs for candidate genes not listed here were obtained from the Ahringer RNAi library [74]. (DOCX 12 kb) Additional file 3: Table S3. Sequences of primers used for quantitative RT-PCR. (DOCX 12 kb)
