# Supplementary material for: Caenorhabditis elegans susceptibility to gut Enterococcus faecalis infection is associated with fat metabolism and epithelial junction integrity
Source: BMC Microbiol. 2016 Jan 15;16:6. doi: 10.1186/s12866-016-0624-8 (PMC4714453; doi:10.1186/s12866-016-0624-8)
Supplement: Additional file 1: Table S1. — C. elegans candidate genes screened via RNAi knockdown. IBD, inflammatory bowel disease; T2D, type 2 diabetes; KD, Kawasaki Disease. (DOCX 15 kb) [file 12866_2016_624_MOESM1_ESM.docx]

**Additional file 1: Table S1:** ***C. elegans* candidate genes screened via RNAi knockdown.** IBD, inflammatory bowel disease; T2D, type 2 diabetes; KD, Kawasaki disease.

| ***C. elegans* gene** | **Human ortholog or related gene** | **Associated human disease** | **Notes** |
| --- | --- | --- | --- |
| *atg-16.1* | ATG16L1 | IBD | Ortholog of the autophagic budding yeast protein Atg16p, and of human ATG16L1 |
| *dlg-1* | DLG5 / DLG1 | IBD | MAGUK (membrane-associated guanylate kinase) protein located at apical adherens junctions in all epithelia |
| *let-653* | MUC3A | IBD | Mucin-like protein believed to provide protective barrier to exposed cellular surfaces |
| *magu-4* | [DLG5](http://asia.ensembl.org/Homo_sapiens/Gene/Summary?g=ENSG00000151208;r=10:79550549-79686378) / DLG1 | IBD | MAGUK protein |
| *pgp-2* | ABCB1 | IBD | ABC transporter localized to gut granule membrane |
| *xbp-1* | XBP-1 | IBD | bZIP transcription factor required for unfolded protein response (UPR) that counteracts cellular stress |
| *glo-1* | RAB32 | IBD / Leprosy | RAB GTPase required for biogenesis of lysosome-related gut granules |
| *lrk-1* | LRRK2 | IBD / Leprosy | Sole *C. elegans* homolog of the leucine-rich repeat kinases LRRK1 and LRRK2; required for polarized localization of synaptic vesicle proteins to presynaptic regions |
| [*nhr-2*](http://www.wormbase.org/species/c_elegans/gene/WBGene00003601?query=nhr-2) | [PPARG](http://asia.ensembl.org/Homo_sapiens/Gene/Summary?g=ENSG00000132170) | IBD / T2D | Nuclear hormone receptor related to mammalian peroxisome proliferator-activated receptors (PPARs) |
| *cyld-1* | CYLD | Leprosy | Human ortholog binds several NF-kB signalling pathway members, can deubiquitinate target proteins |
| *pdr-1* | PARK2 | Leprosy | E3 ubiquitin protein ligase (parkin) |
| [*cdf-2*](http://www.wormbase.org/species/c_elegans/gene/WBGene00011821) | SLC30A8 | T2D | Solute carrier family (zinc transporter) |
| *mrp-5* | ABCC8 / CFTR | T2D | ABC transporter |
| *nhr-35* | HNF4A | T2D | Nuclear hormone receptor related to the mammalian hepatocyte nuclear factor 4 (HNF4) family; expressed exclusively in intestine |
| *nhr-49* | [HNF4A](http://asia.ensembl.org/Homo_sapiens/Gene/Summary?g=ENSG00000101076) | T2D | Nuclear hormone receptor related to the mammalian hepatocyte nuclear factor 4 (HNF4) family |
| *ttm-1* | SLC30A8 | T2D | Solute carrier family (zinc transporter) |
| *num-1* | NUMB | KD | Required for recycling of a transmembrane endocytosis marker |
