# Supplementary material for: Caenorhabditis elegans susceptibility to gut Enterococcus faecalis infection is associated with fat metabolism and epithelial junction integrity
Source: BMC Microbiol. 2016 Jan 15;16:6. doi: 10.1186/s12866-016-0624-8 (PMC4714453; doi:10.1186/s12866-016-0624-8)
Supplement: Additional file 2: Table S2. — Sequences of primers used for PCR amplification of gene-specific amplicons for cloning into the L4440 RNAi vector. RNAi constructs for candidate genes not listed here were obtained from the Ahringer RNAi library [74]. (DOCX 12 kb) [file 12866_2016_624_MOESM2_ESM.docx]

**Additional file 2: Table S2: Sequences of primers used for PCR amplification of gene-specific amplicons for cloning into the L4440** **RNAi vector.** RNAi constructs for candidate genes not listed here were obtained from the Ahringer RNAi library.

| **Gene ID** | **Gene name** | **Primer name** | **Primer sequence** |
| --- | --- | --- | --- |
| C34G6.4 | pgp-2 | ds_pgp-2_F | CAGCATTTGGGGGTGTAAGT |
| C34G6.4 | pgp-2 | ds_pgp-2_R | AGAGAAGCAGCAAGACGAGC |
| Y39E4A.2 | ttm-1 | ds_ttm-1_F | CAACATGGCTCAACTTTTACCTC |
| Y39E4A.2 | ttm-1 | ds_ttm-1_R | AAACTCCAAAATGAGAAGCCACT |
| F14F4.3 | mrp-5 | ds_mrp-5_F | CCCCACTCTACGATCACGTT |
| F14F4.3 | mrp-5 | ds_mrp-5_R | GTTGTCGAGACGATGAGCAA |
| C25F6.2 | dlg-1 | ds_dlg-1_F | GAGGAGCTACGCACAAACCT |
| C25F6.2 | dlg-1 | ds_dlg-1_R | GTTGTTTCCCGCCATGTTCC |
| K08E3.7 | pdr-1 | ds_pdr-1_F | AGATGTGGAAAAGCTCACCG |
| K08E3.7 | pdr-1 | ds_pdr-1_F | ATTCAGCGAAAAGACCTGGA |
| R07B1.12 | glo-1 | ds_glo-1_F | CCTGTCGACGAGAACACAGT |
| R07B1.12 | glo-1 | ds_glo-1_R | CTTTCCTTTGCCGAAGTGCG |
| T03D8.1 | num-1 | ds_num-1_F | GAAGGATATCGAAGCCGAGA |
| T03D8.1 | num-1 | ds_num-1_R | CAGTTCAAATTGATTCCATCG |
| F26D11.11 | let-413 | ds_let-413 F | CTCCTGCTTCGTGAGTGTGT |
| F26D11.11 | let-413 | ds_let-413 R | CATTCAGGCTTGGCTCCTGA |
| F28F8.2 | acs-2 | ds_acs-2 F | TGAGAAATGCACCACGATGT |
| F28F8.2 | acs-2 | ds_acs-2 R | TTGTGAGAGGGAATTCGGAC |
