# Supplementary material for: Caenorhabditis elegans susceptibility to gut Enterococcus faecalis infection is associated with fat metabolism and epithelial junction integrity
Source: BMC Microbiol. 2016 Jan 15;16:6. doi: 10.1186/s12866-016-0624-8 (PMC4714453; doi:10.1186/s12866-016-0624-8)
Supplement: Additional file 3: Table S3. — Sequences of primers used for quantitative RT-PCR. (DOCX 12 kb) [file 12866_2016_624_MOESM3_ESM.docx]

**Additional file 3: Table S3: Sequences of primers used for quantitative RT-PCR.**

| **Gene ID** | **Gene name** | **Primer name** | **Primer sequence** |
| --- | --- | --- | --- |
| K10C3.6 | nhr-49 | RT_nhr-49_F | CTGCAACGGGTGTAAGGGAT |
| K10C3.6 | nhr-49 | RT_nhr-49_R | ACTGGCTCCAGATGTTGGTG |
| C25F6.2 | dlg-1 | RT_dlg-1_F | ATCATTGAGGAGGGAGCAGC |
| C25F6.2 | dlg-1 | RT_dlg-1_R | AGTGAATTGAGAACATTGACCGC |
| F10D2.9 | fat-7 | RT_fat-7_F | GTGGCTCTCTTTGCTGCTCT |
| F10D2.9 | fat-7 | RT_fat-7_R | GACGGCCGTTTGCCATTTAG |
| F28F8.2 | acs-2 | RT_acs-2_F | TGACGTGCTCAAGTCTCCAC |
| F28F8.2 | acs-2 | RT_acs-2_R | CATGGGGCTCCTCCGATAAC |
| C29F3.1 | ech-1 | RT_ech-1_F | AACTGCTGCTCAGCTAGGAC |
| C29F3.1 | ech-1 | RT_ech-1_R | TTAGGCATCGGACGACGAAG |
| C25A11.4 | ajm-1 | RT_ajm-1_F | AAAGGAGAAAGCCGAGACGG |
| C25A11.4 | ajm-1 | RT_ajm-1_R | CGTTCACGCTCGATTCGTTC |
| F26D11.11 | let-413 | RT_let-413_F | GGTTCATTGACGCTCCTGCT |
| F26D11.11 | let-413 | RT_let-413_R | TGCATGTTTTGAGTGCCTGC |
| T04C12.6 | act-1 | RT_act-1_F | CCCCACTCAATCCAAAGGCT |
| T04C12.6 | act-1 | RT_act-1_R | GTACGTCCGGAAGCGTAGAG |
| F36A4.7 | ama-1 | RT_ama-1_F | GGAGCTCGAGTGGATCTTCG |
| F36A4.7 | ama-1 | RT_ama-1_R | TTGTGGAGAGTCGGTTGACG |
| C01H6.5 | nhr-23 | RT_nhr-23_F | TGCCGCTGGACTTATTCTGG |
| C01H6.5 | nhr-23 | RT_nhr-23_R | GCAATCGTCGCAGTGTCAAG |
